# Supplementary material for: Dynamic metastable polymersomes enable continuous flow manufacturing
Source: Nat Commun. 2023 Oct 6;14:6237. doi: 10.1038/s41467-023-41883-6 (PMC10558441; doi:10.1038/s41467-023-41883-6)
Supplement: Supplementary file 1 — Supplementary Information [file 41467_2023_41883_MOESM1_ESM.pdf]

Supplementary Information

Dynamic metastable polymersomes enable continuous flow manufacturing

Chin Ken Wong,*[1] Rebecca Y. Lai,[1] Martina H. Stenzel*[1]

[1]*School of Chemistry, The University of New South Wales (UNSW), Sydney NSW, Australia*

**Correspondence to:* c.kenwong@unsw.edu.au and m.stenzel@unsw.edu.au

Supplementary Figures

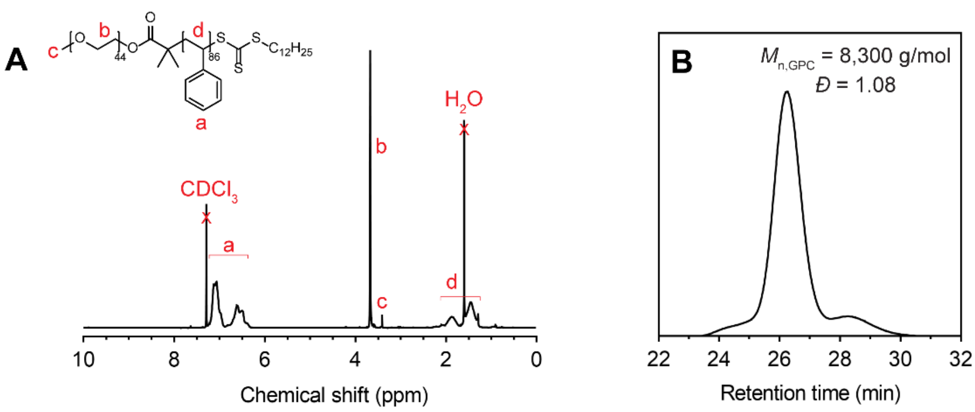

Supplementary Figure 1. (A) ^1H NMR spectrum (400 MHz, $CDCl_3$) and (B) DMF GPC trace of PEO_{44}-*b*-PS_{86}. The small shoulder observed at 28.5 min corresponds to unreacted PEO_{44} left behind during PEO_{44}-DDMAT macroRAFT agent synthesis.

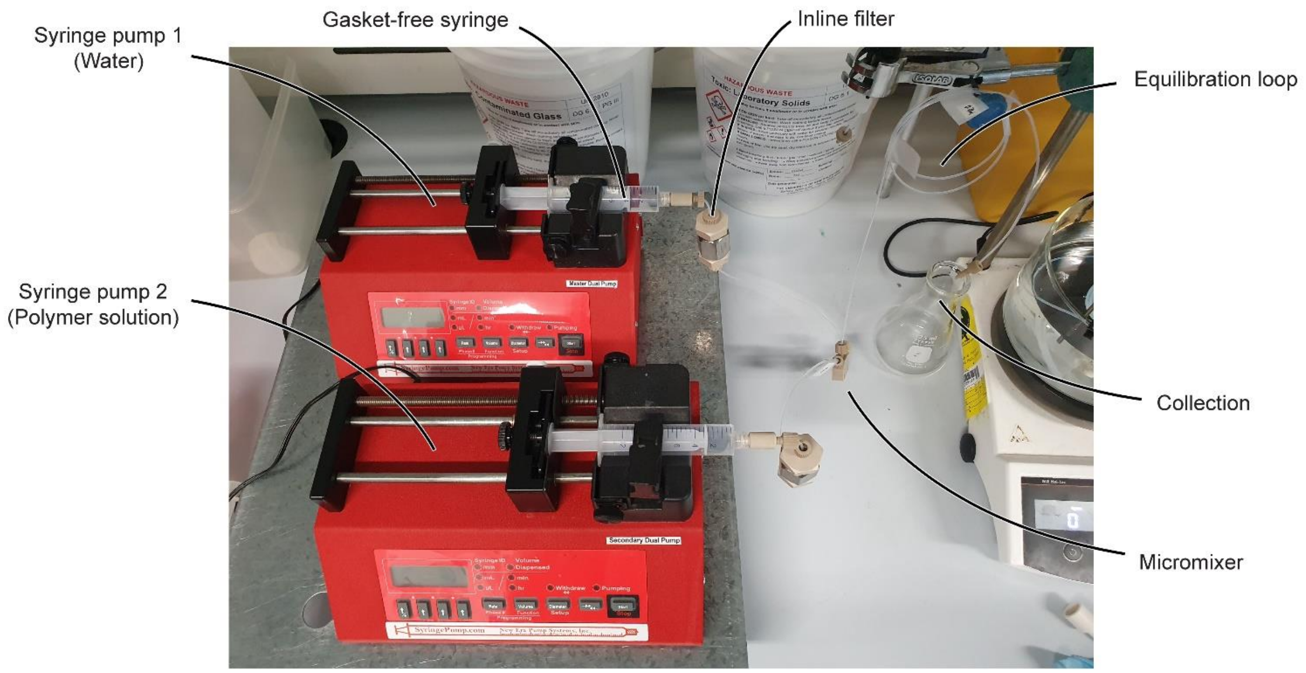

Supplementary Figure 2. Photograph of the setup used for continuous flow self-assembly.

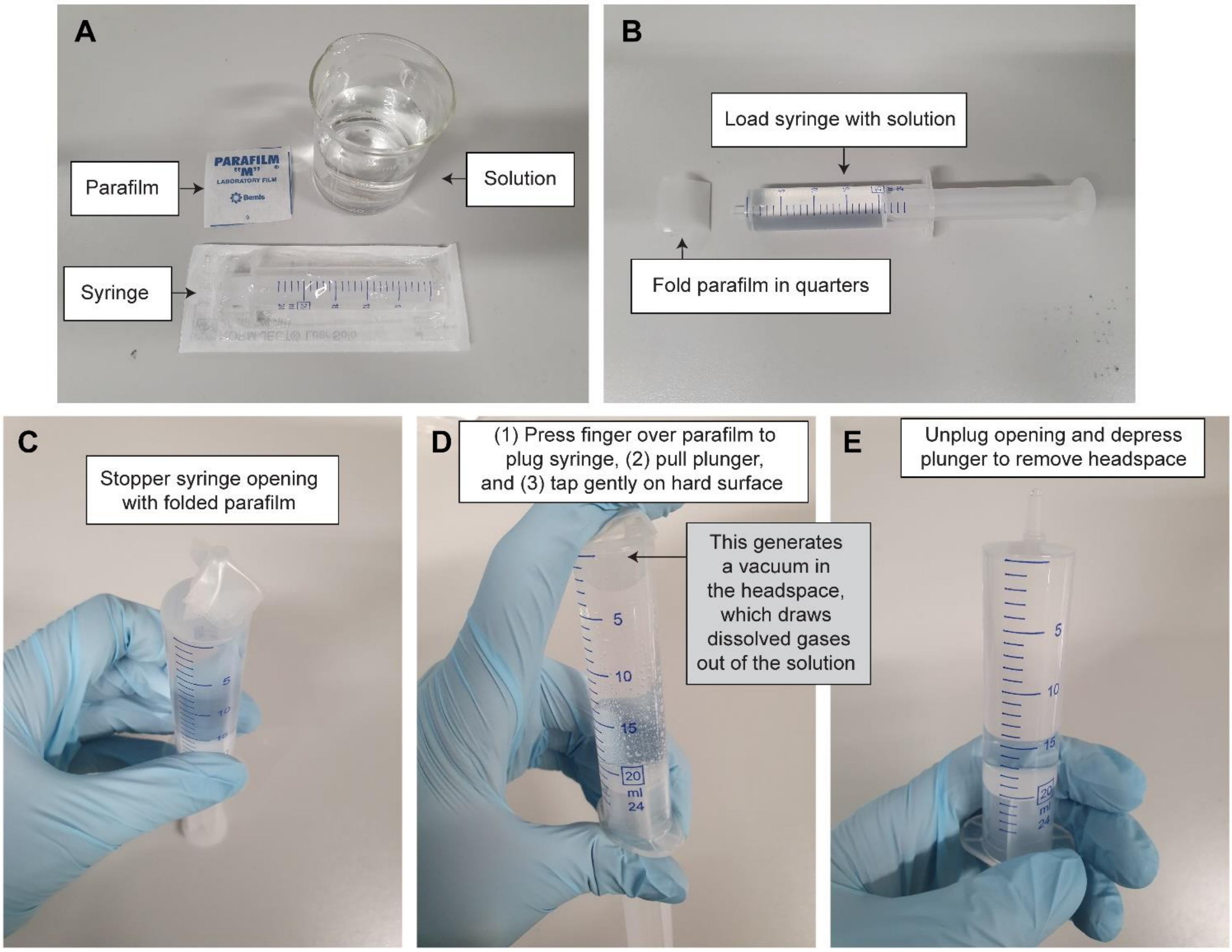

Supplementary Figure 3. (A-E) A series of photographs outlining the degassing procedure used. The process was repeated at least five times to ensure proper degassing.

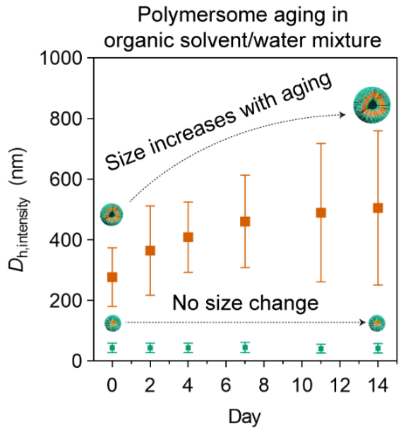

Supplementary Figure 4. DLS data showing polymersomes generated at $Q_{organic}/Q_{total}$ = 0.7 (Pathway 2) increase in size with aging for 7 days before plateauing, while micelles generated at $Q_{organic}/Q_{total}$ = 0.1 (Pathway 1) retain their size even after aging for 14 days. Intensity-averaged data are expressed in mean ± SD (n = 3). All samples were analyzed in their respective organic solvent/water mixtures.

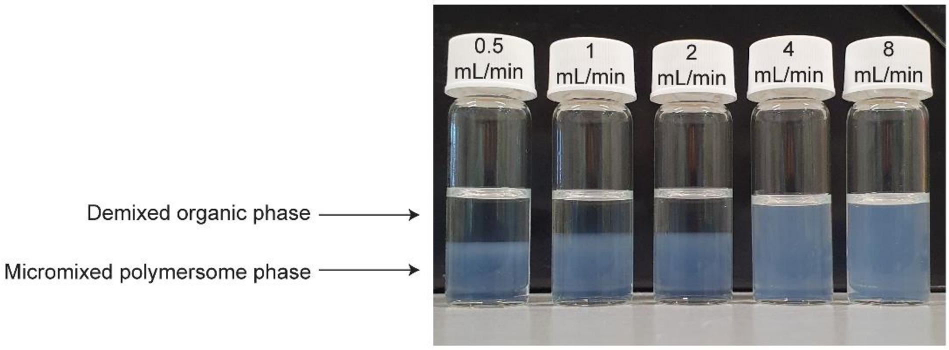

Supplementary Figure 5. Photograph of solutions obtained using the continuous flow setup in Supplementary Figure 2 at flow rates of 0.5, 1, 2, 4 and 8 mL/min. For all flow rates tested, we confirmed the occurrence of polymersome formation by TEM analysis. However, at lower flow rates (0.5-2 mL/min), the polymersome phase, which displays a blue tint due to light scattering, is demixed from the organic phase, presumably due to the peculiar miscibility gap between THF and water. The interface between the two phases is highly diffuse, and if left to sit undisturbed, would gradually mix over time. We found that a gentle swirl or quick flip of the vial is sufficient to homogenize these demixed solutions without significantly compromising polymersome quality. At higher flow rates (4-8 mL/min), the micromixing efficiency is sufficiently high as to allow immediate homogenization within the mixing chamber of the micromixer.

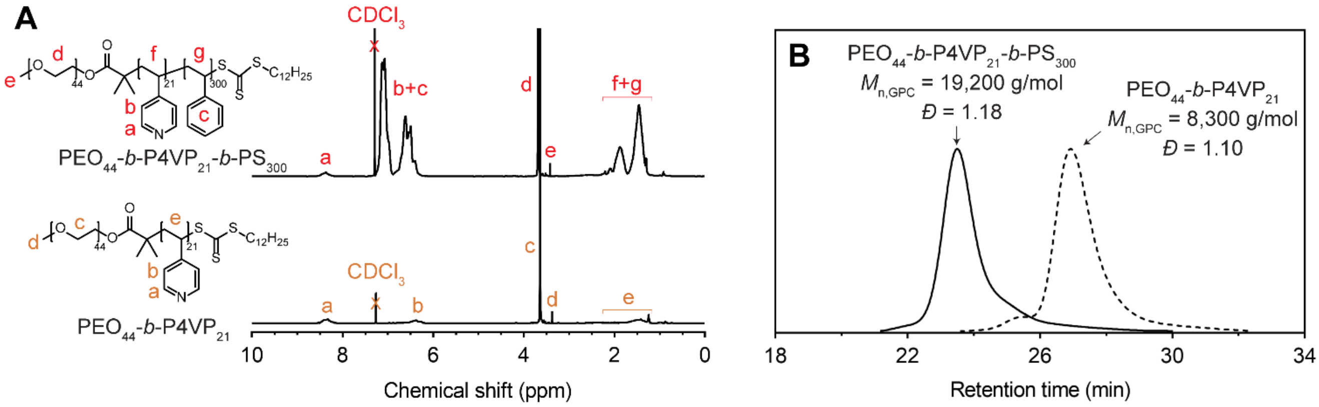

Supplementary Figure 6. (A) ^{1}H NMR spectra (400 MHz, $CDCl_3$) and (B) DMF GPC traces of PEO_{44}-*b*-$P4VP_{21}$ and PEO_{44}-*b*-$P4VP_{21}$-*b*-PS_{300}.

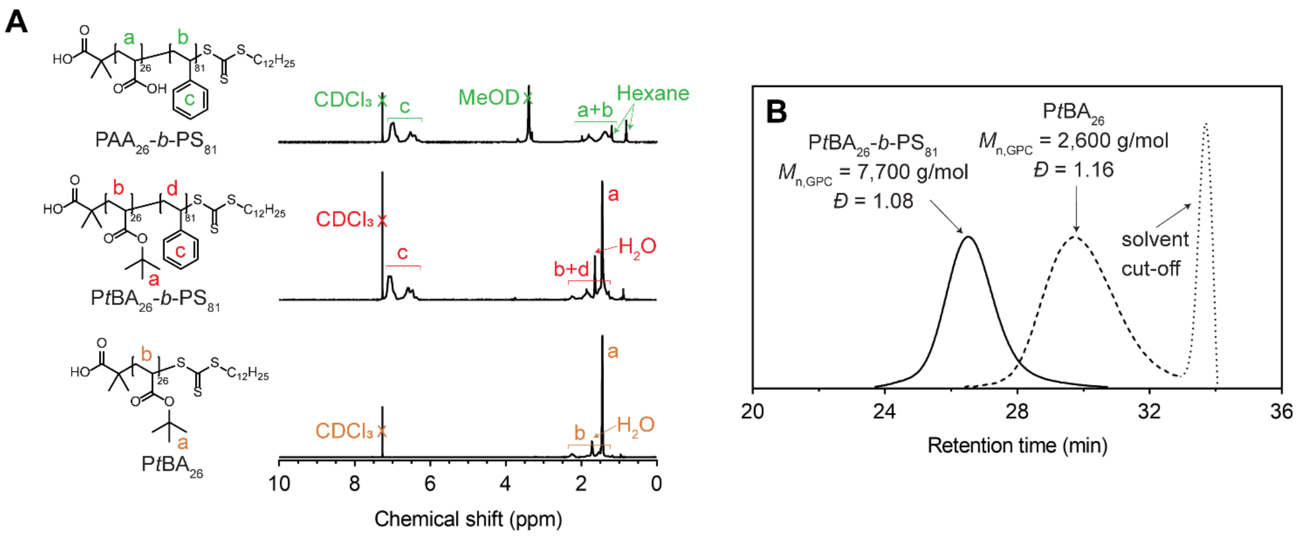

Supplementary Figure 7. (A) ^{1}H NMR spectra (400 MHz) of PtBA$_{26}$ in $CDCl_3$, PtBA$_{26}$-*b*-PS$_{81}$ in $CDCl_3$, and PAA$_{26}$-*b*-PS$_{81}$ in 1:5 (v/v) MeOD/$CDCl_3$. (B) DMF GPC traces of PtBA$_{26}$ and PtBA$_{26}$-*b*-PS$_{81}$. PAA$_{26}$-*b*-PS$_{81}$ was not analyzed by GPC because PAA strongly interacts with the GPC column media.

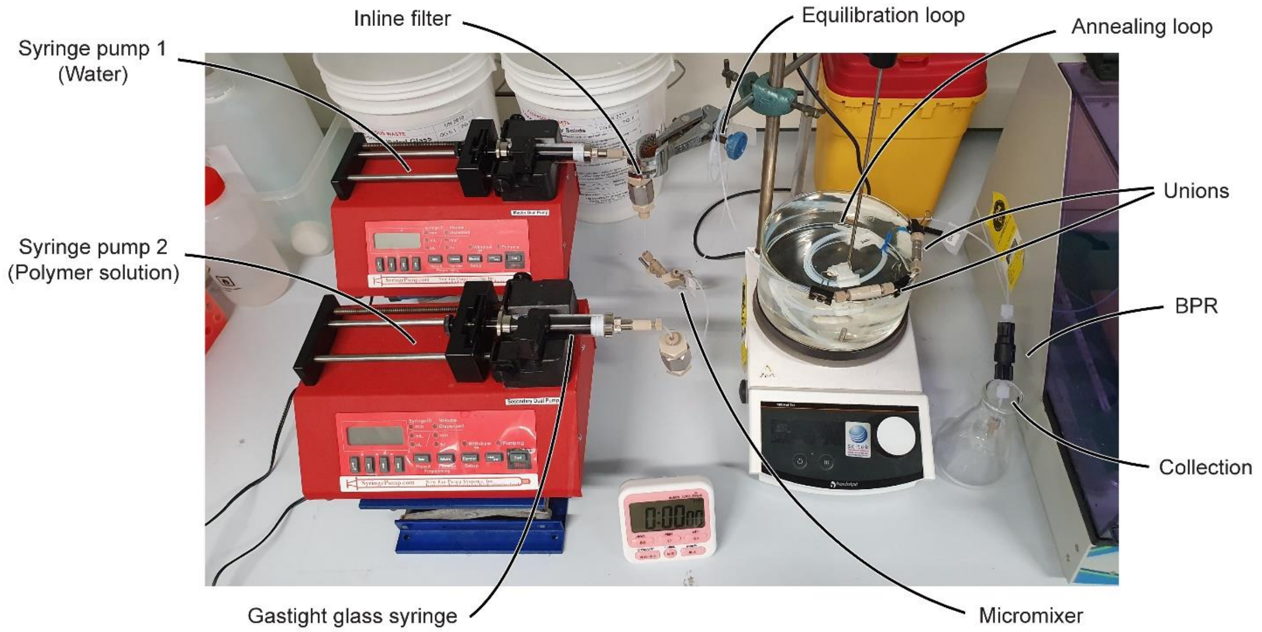

Supplementary Figure 8. Photograph of setup used to perform continuous flow self-assembly and downstream annealing.

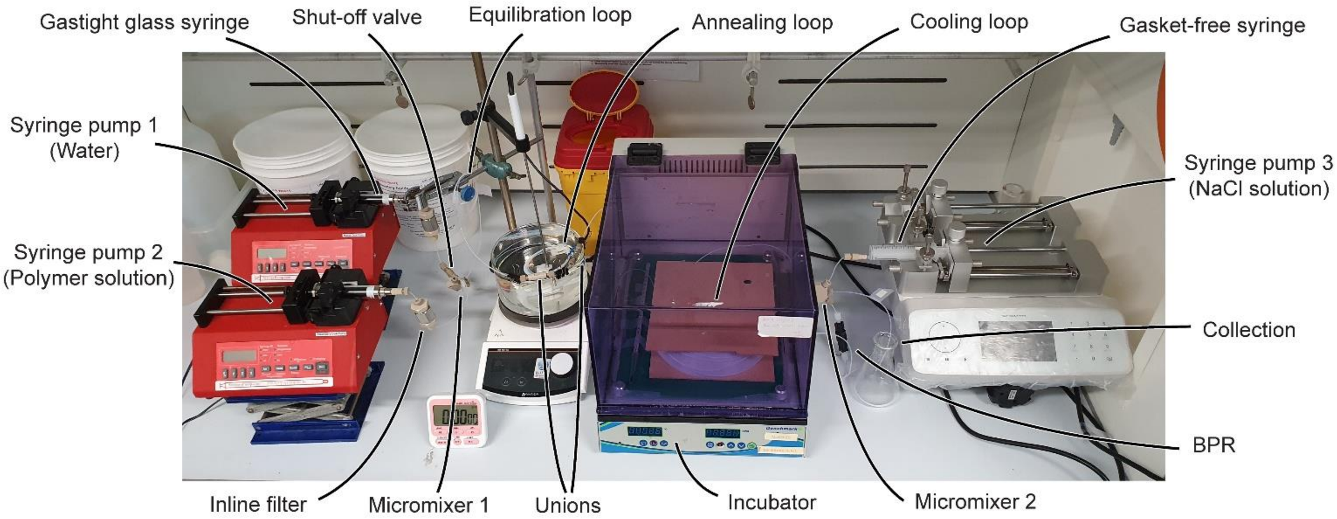

Supplementary Figure 9. Photograph of setup used to perform continuous flow self-assembly, downstream annealing, cooling, and shape transformation.

Supplementary Tables

Supplementary Table 1. Summary of DLS data presented in Figures 1C.

$Q_{organic}/Q_{total}$	$Q_{organic}$	Q_{water}	$D_{h,intensity}$ (nm)[1]	PDI
0.1	0.1	0.9	43 ± 16	0.145 ± 0.004
0.2	0.2	0.8	41 ± 14	0.129 ± 0.014
0.3	0.3	0.7	49 ± 19	0.131 ± 0.011
0.4	0.4	0.6	90 ± 39	0.158 ± 0.014
0.5	0.5	0.5	127 ± 51	0.134 ± 0.015
0.6	0.6	0.4	181 ± 69	0.130 ± 0.007
0.7	0.7	0.3	277 ± 97	0.120 ± 0.007

[1]Samples were analyzed in their respective organic solvent/water mixtures.

Supplementary Table 2. Summary of DLS data presented in Figures 2C.

Day	$D_{h,intensity}$ (nm)	PDI
0	125 ± 46	0.145 ± 0.020
2	152 ± 51	0.126 ± 0.018
4	160 ± 49	0.080 ± 0.022
7	192 ± 67	0.138 ± 0.009
11	193 ± 63	0.096 ± 0.035
14	195 ± 65	0.095 ± 0.003

Supplementary Table 3. Summary of DLS data presented in Figures 3A.

$C_{polymer}$ (mg/mL)	$D_{h,intensity}$ (nm)	PDI
1	125 ± 46	0.145 ± 0.020
5	146 ± 44	0.089 ± 0.009
9	170 ± 49	0.078 ± 0.008

Supplementary Table 4. Summary of DLS data presented in Figures 3B.

Q_{total} (mL/min)	$D_{h,intensity}$ (nm)	PDI
0.5	138 ± 51	0.225 ± 0.020
1	125 ± 46	0.145 ± 0.020
2	122 ± 39	0.118 ± 0.018
4	110 ± 34	0.089 ± 0.008
8	107 ± 27	0.045 ± 0.015

Supplementary Table 5. Summary of DLS data presented in Figures 3E(iv).

Day	$D_{h,intensity}$ (nm)	PDI
0	136 ± 46	0.141 ± 0.007
2	161 ± 47	0.148 ± 0.060
4	170 ± 54	0.101 ± 0.009
7	170 ± 54	0.077 ± 0.027

Supplementary Table 6. Summary of DLS data presented in Figures 3F(iv).

Day	$D_{h,intensity}$ (nm)	PDI
0	83 ± 30	0.138 ± 0.007
2	100 ± 29	0.073 ± 0.021
4	102 ± 31	0.088 ± 0.006
7	104 ± 30	0.075 ± 0.025

Supplementary Table 7. Summary of DLS data presented in Figures 4B(iv).

Temperature (°C)	$D_{h,intensity}$ (nm)	PDI
20	97 ± 27	0.064 ± 0.007
30	100 ± 28	0.071 ± 0.014
40	115 ± 27	0.026 ± 0.015
50	150 ± 36	0.041 ± 0.021
60	176 ± 42	0.030 ± 0.015
70	190 ± 53	0.061 ± 0.010

Supplementary Methods

Chemicals and materials

All chemicals (reagent grade) were purchased from Sigma-Aldrich (Australia) and used as received unless otherwise mentioned. Tetrahydrofuran (THF, HPLC grade) and 1,4-dioxane (analytical grade) were purchased from ChemSupply (Australia). Carbon-coated copper TEM grids (300 mesh) were purchased from ProSciTech (Australia). Styrene (99%), 4-vinylpyridine (4VP, 95%) and *tert*-butyl acrylate (tBA, 98%) were deinhibited by passing through a basic alumina plug prior to use. AIBN was recrystallized from methanol prior to use. The polyethylene oxide-based macroRAFT agent (PEO_{44}-DDMAT) was synthesized according to our earlier report.[1] Polytetrafluoroethylene (PTFE) syringe filters (0.45 µm, 33 mm) were purchased from Grace Davison Discovery Sciences (Australia). Polyethersulfone (PES) syringe filters (0.45 µm, 33 mm) were purchased from Merck Millipore Ltd (Ireland). Cellu·Sep® regenerated cellulose dialysis tubing with 3.5 kDa molecular weight cut-off (MWCO) was purchased from Adelab Scientific (Australia). A list of continuous flow components used is provided below:

Flow accessory name	Schematic representation	Photograph	Supplier	Part number	Material
Syringe pump	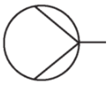	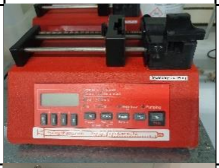	New Era	NE-1010X	N/A
Syringe pump	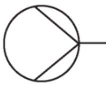	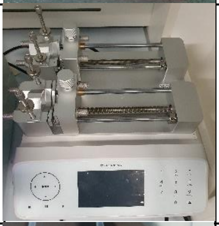	Chemyx	Fusion 4000-X	N/A
Micromixer	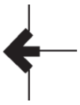	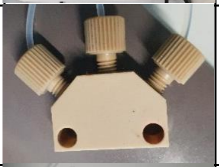	Vici-Jour	JR-9000-0670	PEEK
PTFE tubing - 1/16" OD, 1.0 mm ID	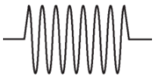	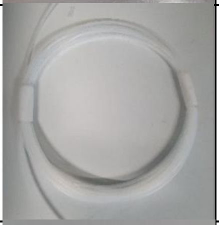	Vici-Jour	JR-T-6807	PTFE
Standard fingertight fitting for 1/16" OD tubing, 10-32 threads	N/A	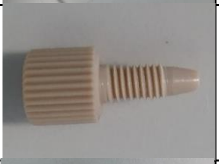	Yika Technology	FJ-101	PEEK
Short Fingertight fitting for 1/16" OD tubing, 10-32 threads	N/A	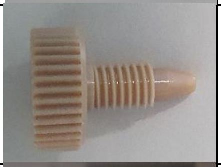	Yika Technology	FJ-102	PEEK
10-32 female to adapter	N/A	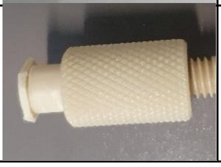	Vici-Jour	JR-0623	PTFE

Union for 1/16" OD tubing	N/A	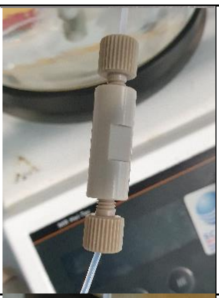	Yika Technology	CT-102	PEEK
Union for 1/16" OD tubing	N/A	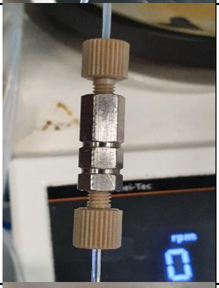	Shimadzu	228-16004-13	Stainless steel
Inline semi-prep filter assembly, 10 µm PEEK frit	N/A	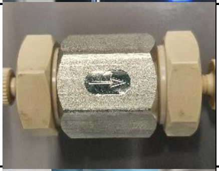	IDEX-HS	A-411	PTFE
Backpressure regulator (BPR), 100 psi	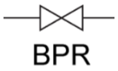	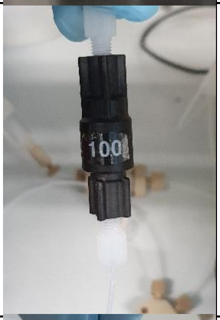	IDEX-HS	P-787	PEEK
Shut-off valve	N/A	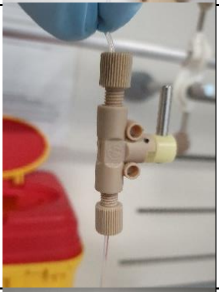	IDEX-HS	P-732A	PEEK
SGE gastight syringe, 10 mL	N/A	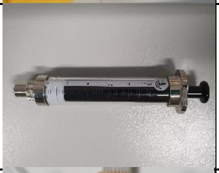	SGE	10MDR-LL-GT	Glass
Secondary micromixer	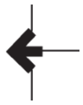	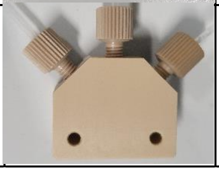	Yika Technology	CT-122	PEEK

Gel permeation chromatography (GPC)

Polymer molecular weight and dispersity were characterized on a Shimadzu modular system equipped with DGU-12A degasser, LC-10AT pump, SIL-10AD auto injector, CTO-10A column oven, RID-10A differential

refractive index detector, and three Phenomenex 5.0 µm bead-size columns connected in series (10^5, 10^4 and 10^3 Å). Dimethylformamide (DMF) containing 0.1% lithium bromide (LiBr) was used as the GPC eluent. The sample (~1 mg/mL) was filtered through a 0.45 µm PTFE syringe filter prior to injection. Number-average molecular weight ($M_{n,GPC}$) was estimated based on a narrow molecular weight (100 to 1 × 10^6 g/mol) poly(methyl methacrylate) (PMMA) calibration standard. Note that the $M_{n,GPC(DMF)}$ values provided herein for all PS-containing block copolymers are underestimates because PS chains are known to adopt a reduced chain conformation in DMF (Hildebrand solubility parameters: δ_{PS} =16.6–20.2 $MPa^{-1/2}$ and δ_{DMF} = 24.7 $MPa^{-1/2}$).[2] These block copolymers therefore exhibit increased apparent retention times in GPC and thus have smaller apparent $M_{n,GPC(DMF)}$ values.

Transmission electron microscopy (TEM)

In a typical sample preparation, a 6 µL droplet of particle solution (<0.5 mg/mL) was placed onto a carbon-coated copper grid (300 mesh size) and allowed to sit for at least 10 min. A pre-cut filter paper was then used to blot the droplet, leaving behind a thin film. The sample was then left to evaporate under a benchtop fume exhaust arm. No staining was used. TEM analyses were performed either on an FEI Tecnai G2 20 TEM (120 kV) equipped with a BM Eagle 2K CCD Camera or a JEOL TEM-1400 (80 kV) equipped with an EMSIS Phurona CMOS Camera. Images were processed and analyzed with ImageJ.[3]

Dynamic light scattering (DLS)

DLS measurements were conducted on a Zetasizer Nano ZSP instrument. Samples were measured at a backscatter angle of 173° at 25 °C. Samples in organic solvent/water mixtures were measured undiluted (concentrations between 0.1-0.7 mg/mL) in a 600 µL quartz cuvette, while aqueous samples were measured at a concentration of 0.1 mg/mL in disposable 3 mL PMMA cuvettes.

Turbidity assay

Turbidity measurements were conducted on a Varian Cary® 50 UV-Vis spectrophotometer. Samples were measured in a 600 µL quartz cuvette with 0.2 cm pathlength (Thorlabs, Germany). For the turbidity (aging) experiment in Figure 2B(i) and 2B(ii), we first prepared a parent polymersome solution on day 0 using the setup shown in Supplementary Figure 2 and the following flow conditions: (i) $Q_{organic}/Q_{total}$ = 0.7, (ii) Q_{total} = 1 mL/min and (iii) $c_{polymer}$ = 1 mg/mL. We then removed aliquots from the parent polymersome solution for analysis on days 0, 2, 4, 7, 11 and 14. For the turbidity (annealing) experiment in Figure 5B, we first prepared an annealed polymersome solution using the setup shown in Supplementary Figure 8 and following flow

conditions: (i) $Q_{organic}/Q_{total}$ = 0.7, (ii) Q_{total} = 4 mL/min, (iii) $c_{polymer}$ = 1 mg/mL and (iv) $T_{annealing}$ = 70 °C. The sample was collected directly into a cuvette and immediately analyzed at 6 s intervals for a total of 5 min.

RAFT polymerization of polyethylene oxide-*block*-polystyrene (PEO$_{44}$-*b*-PS$_{86}$)

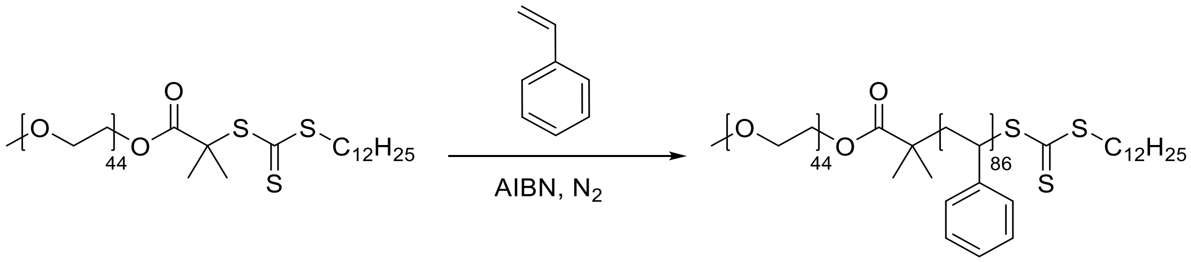

The polymerization was carried out in bulk using the following stoichiometry of [styrene]:[PEO$_{44}$-DDMAT]:[AIBN] = [200]:[1]:[0.1]. To a solution of styrene (4.40 g, 4.84 mL, 42.2 mmol) was added PEO$_{44}$-DDMAT (0.50 g, 0.211 mmol) and AIBN (3.47 mg, 21.1 µmol). The reaction mixture was degassed for 15 min with nitrogen gas over an ice bath to prevent monomer evaporation. The polymerization was initiated by submerging the reaction vessel into a pre-heated oil bath at 65 °C. After 36 h, the polymerization was quenched by exposing the reaction mixture to air and submerging the reaction mixture in an ice bath. The crude product was diluted in THF and purified by precipitation into hexane. The purification process was repeated 3 times in total. The resulting precipitate was then dried overnight in a vacuum oven at 40 °C to yield PEO$_{44}$-*b*-PS$_{86}$ as a yellow powder. %Conversion = 43%; $M_{n,NMR}$ = 11,260 g/mol; $M_{n,GPC(DMF)}$ = 8,300 g/mol; $Đ$ = 1.08.

RAFT polymerization of polyethylene oxide-*block*-poly(4-vinylpyridine)-*block*-polystyrene (PEO$_{44}$-*b*-P4VP$_{21}$)

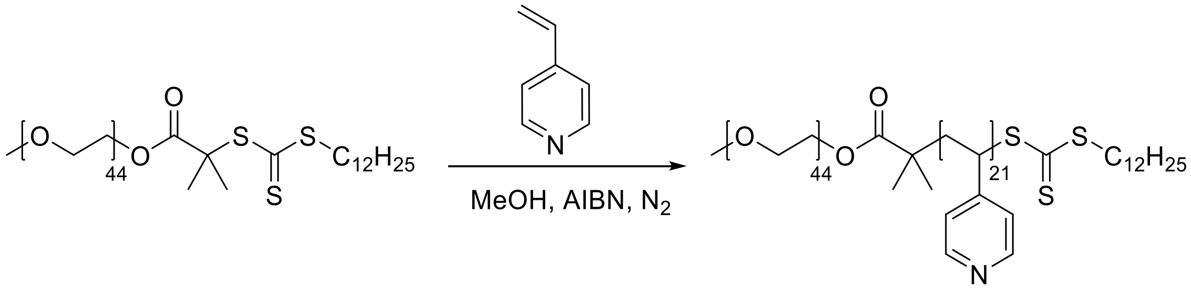

The polymerization was carried out in methanol using the following stoichiometry of [4-vinylpyridine]:[PEO$_{44}$-DDMAT]:[AIBN] = [25]:[1]:[0.1]. To a solution of 4-vinylpyridine (1.00 g, 9.51 mmol) was added methanol (293 µL), PEO$_{44}$-DDMAT (899 mg, 0.38 mmol) and AIBN (6.25 mg, 38.0 µmol). The reaction mixture was degassed for 15 min with nitrogen gas over an ice bath to prevent monomer evaporation. The polymerization was initiated by submerging the reaction vessel into a pre-heated oil bath at 65 °C. After 22 h 20 min, the polymerization was quenched by exposing the reaction mixture to air and

submerging the reaction mixture in an ice bath. The crude product was diluted in DMF and purified by precipitation into 2:8 (v/v) diethyl ether/hexane. The purification process was repeated 3 times in total. The resulting precipitate was then dried overnight in a vacuum oven at 40 °C to yield PEO_{44}-*b*-$P4VP_{21}$ as a yellow powder. %Conversion = 84%; $M_{n,NMR}$ = 4,570 g/mol; $M_{n,GPC(DMF)}$ = 6,100 g/mol; $Đ$ = 1.10.

RAFT polymerization of polyethylene oxide-*block*-poly(4-vinylpyridine)-*block*-polystyrene (PEO_{44}-*b*-$P4VP_{21}$-*b*-PS_{300})

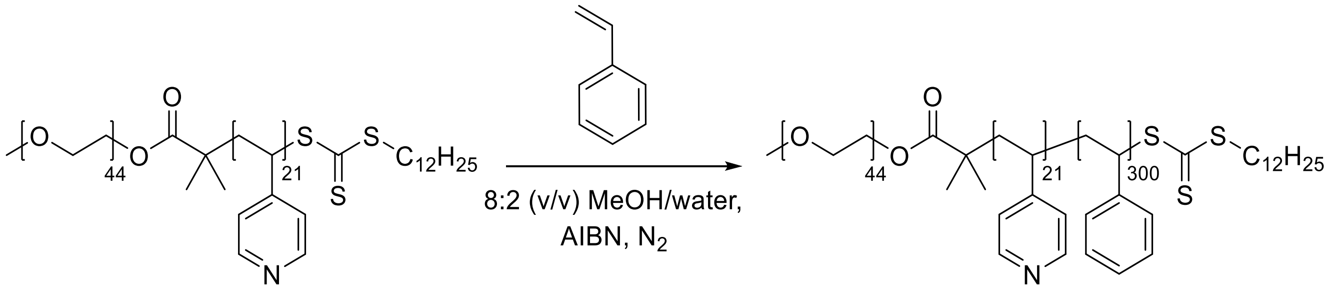

The polymerization was carried out under dispersion conditions (25 wt% solids content) using the following stoichiometry of [styrene]:[PEO_{44}-*b*-$P4VP_{21}$]:[AIBN] = [300]:[1]:[0.1]. To a solution of styrene (683 mg, 6.56 mmol) was added methanol (1.88 mL), water (470 µL), PEO_{44}-*b*-$P4VP_{21}$ (100 mg, 21.9 µmol) and AIBN (0.71 mg, 4.37 µmol). The reaction mixture was degassed for 20 min with nitrogen gas over an ice bath to prevent monomer evaporation. The polymerization was initiated by submerging the reaction vessel into a pre-heated oil bath at 70 °C. After 22 h, the polymerization was quenched by exposing the reaction mixture to air and submerging the reaction mixture in an ice bath. The crude product was purified by dialysis against methanol in a 3.5 kDa MWCO dialysis tubing. The resulting solution was evaporated using a rotary evaporator, and the product dried overnight in a vacuum oven at 40 °C to yield PEO_{44}-*b*-$P4VP_{21}$-*b*-PS_{300} as a yellow powder. %Conversion = >99%; $M_{n,NMR}$ = 35,820 g/mol; $M_{n,GPC(DMF)}$ = 19,200 g/mol; $Đ$ = 1.18.

RAFT polymerization of poly(*tert*-butyl acrylate) ($PtBA_{26}$)

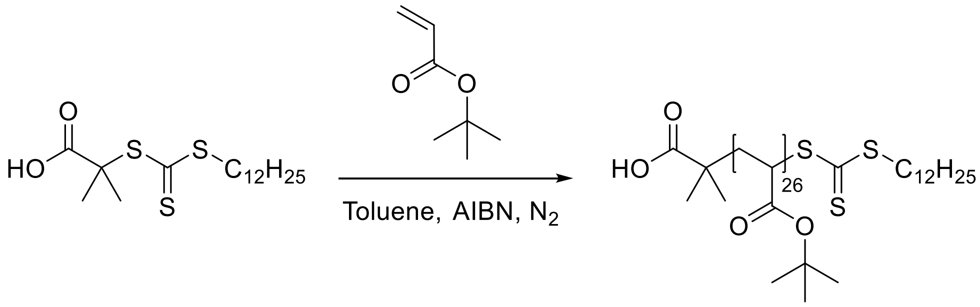

The polymerization was carried out in toluene using the following stoichiometry of [tBA]:[DDMAT]:[AIBN] = [50]:[1]:[0.1]. To a solution of tBA (1.75 g, 1.37 mL, 0.014 mol) in toluene (277 µL) was added DDMAT

(99.6 mg, 0.273 mmol) and AIBN (4.50 mg, 27.3 µmol). The reaction mixture was degassed for 20 min with nitrogen gas over an ice bath to prevent monomer evaporation. The polymerization was initiated by submerging the reaction vessel into a pre-heated oil bath at 60 °C. After 1 h 50 min, the polymerization was quenched by exposing the reaction mixture to air and submerging the reaction mixture in an ice bath. The solvent (toluene) and unreacted monomer were removed by reconstituting the crude polymer in toluene (10 mL) and evaporating to dryness for at least three times. The purified polymer was then dried overnight in a vacuum oven at 40 °C to yield $PtBA_{26}$ as a yellow viscous liquid. %Conversion = 52%; $M_{n,NMR}$ = 3,700 g/mol; $M_{n,GPC(DMF)}$ = 2,600 g/mol; $Đ$ = 1.16.

RAFT polymerization of poly(*tert*-butyl acrylate)-*block*-polystyrene ($PtBA_{26}$-*b*-PS_{81})

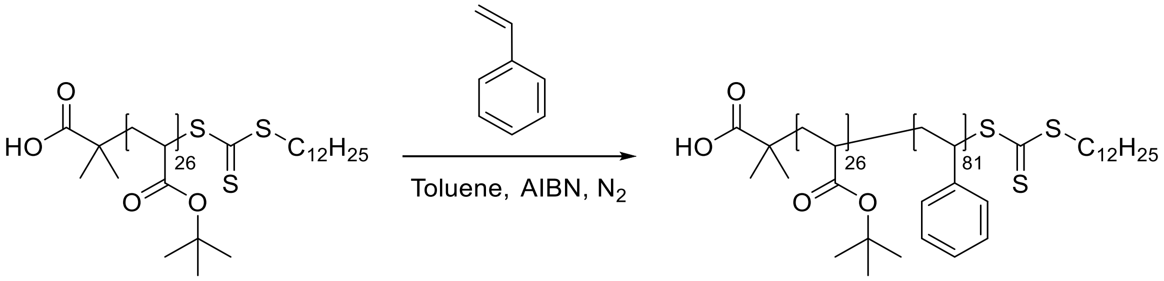

The polymerization was carried out using the following stoichiometry of [styrene]:[$PtBA_{26}$-DDMAT]:[AIBN] = [300]:[1]:[0.1]. To a solution of styrene (3.38 g, 3.72 mL, 0.032 mmol) and toluene (4.64 mL) was added $PtBA_{26}$-DDMAT (400 mg, 0.108 mmol) and AIBN (1.78 mg, 10.8 µmol). The reaction mixture was degassed for 25 min with nitrogen gas over an ice bath to prevent monomer evaporation. The polymerization was initiated by submerging the reaction vessel into a pre-heated oil bath at 65 °C. After 24 h, the polymerization was quenched by exposing the reaction mixture to air and submerging the reaction mixture in an ice bath. The crude product was diluted in THF and purified by precipitation into hexane. The purification process was repeated 3 times in total. The resulting precipitate was then dried overnight in a vacuum oven at 40 °C to yield PEO_{44}-*b*-PS_{86} as a yellow powder. %Conversion = 27%; $M_{n,NMR}$ = 12,140 g/mol; $M_{n,GPC(DMF)}$ = 7,700 g/mol; $Đ$ = 1.08.

Deprotection of poly(*tert*-butyl acrylate)-*block*-polystyrene ($PtBA_{26}$-*b*-PS_{81}) to yield poly(*acrylic acid*)-*block*-polystyrene (PAA_{26}-*b*-PS_{81})

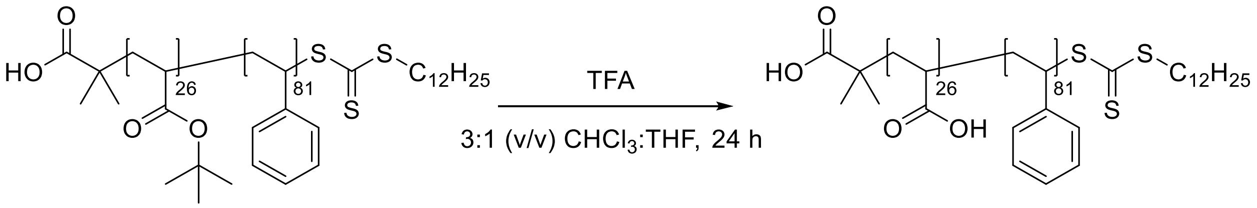

The diblock copolymer PtBA$_{26}$-*b*-PS$_{81}$ (1.00 g, 83.3 µmol) was dissolved in 3:1 (v/v) chloroform:THF (16 mL) and cooled to 0 °C in an ice bath. To the cooled solution was then added trifluoroacetic acid (TFA, 8 mL) dropwise under stirring. The reaction mixture was allowed to warm to room temperature and further stirred for 8 h. The solvent and TFA were removed by evaporation using a gentle stream of nitrogen, redissolved in THF and precipitated into 4:1 (v/v) hexane:diethyl ether. The product was then dried in a vacuum oven at 40 °C to yield PAA$_{26}$-*b*-PS$_{81}$ as a yellow solid. The degree of deprotection was estimated to be >99% by ^{1}H NMR.

Continuous flow self-assembly procedure for PEO$_{44}$-*b*-PS$_{86}$

A 1 mg/mL stock solution of PEO$_{44}$-*b*-PS$_{86}$ in 1:4 (v/v) dioxane/THF was prepared beforehand and stirred in an airtight Schott bottle at 100 rpm until required. The same stock solution was used in all experiments to minimize any concentration errors. Stock solutions with higher concentrations (up to 9 mg/mL) were prepared the same way as above. Whenever needed, the required volume of PEO$_{44}$-*b*-PS$_{86}$ stock solution was removed with a rubber gasket-free syringe and filtered through a 0.45 µm PTFE syringe filter to remove dust. Note that the filtration step is crucial as prolonged/repeated use of unfiltered stock solutions may lead to eventual clogging of the micromixer. As a secondary precaution to prevent clogging, we place 10 µm inline filters before the micromixer in our flow setup (see Supplementary Figure 2). The inline filters can be replaced with Luer lock syringe filters although this is not recommended when using high flow rates or when the flow system is operating under a large backpressure. The filtered PEO$_{44}$-*b*-PS$_{86}$ solution was then loaded into a rubber gasket-free syringe and degassed at least 3 times following the procedure in Supplementary Figure 3 before mounting the syringe onto a syringe pump. The water used in all experiments was obtained directly from a Milli-Q dispenser and filtered through a 0.45 µm PES syringe filter prior to use. The required volume of water was loaded into a syringe and similarly degassed following the procedure in Supplementary Figure 3 for at least 5 times before mounting the syringe onto another syringe pump. Both syringes (one containing the organic phase and the other containing the aqueous phase) were then connected to the micromixer as shown in Supplementary Figure 2 to complete the continuous flow setup. Next, the syringe pumps were set to dispense at any required combination of flow rates outlined in Table S1 (see Q_{organic} and Q_{water}). Once dispensing has begun, we typically allow the system to equilibrate for at least four times the volume of the equilibration loop (4 × 0.71 mL = 2.84 mL). After sufficient equilibration, the resulting mixture was collected directly into a glass vial and gently swirled to ensure homogeneity. The sample was then either analyzed directly, allowed to age undisturbed, or quenched by extensive dialysis against water in a 3.5 kDa MWCO dialysis tubing.

Shape transformation of PEO_{44}-*b*-PS_{86} polymersomes

A fresh batch of polymersome solution was prepared by continuous flow self-assembly using the following flow conditions: Q_{total} = 1 mL/min, $Q_{organic}$ = 0.7 mL/min, Q_{water} = 0.3 mL/min and $c_{polymer}$ = 1 mg/mL. The solution was aged for 14 days before subjection to shape transformation via osmotic pressure. Osmotic pressure was induced by adjusting the salinity of the aged polymersome solution to 50 mM NaCl using a stock solution of 5 M NaCl. The solution was then extensively dialyzed against 50 mM NaCl in a 3.5 kDa MWCO dialysis tubing to remove the organic solvents. The resulting solution was centrifuged at 12,000 rpm (13,201 x *g*) for 10 min to yield an off-white pellet of stomatocytes. The supernatant was carefully removed with a pipette and the pellet redispersed in water (n.b., the supernatant contains a small portion of <150 nm spherical polymersomes that were unaffected by the shape transformation procedure). The centrifugation/redispersion process was repeated three times in order to complete the solvent transfer process and to yield a pure stomatocyte phase for TEM analysis.

Continuous flow self-assembly procedure for PEO_{44}-*b*-$P4VP_{21}$-*b*-PS_{300}

A 1 mg/mL stock solution of PEO_{44}-*b*-$P4VP_{21}$-*b*-PS_{300} in 1:4 (v/v) dioxane/THF was prepared beforehand and stirred in an airtight Schott bottle at 100 rpm until required. The same stock solution was used in all experiments to minimize any concentration errors. Whenever needed, the required volume of PEO_{44}-*b*-$P4VP_{21}$-*b*-PS_{300} stock solution was removed with a rubber gasket-free syringe and filtered through a 0.45 µm PTFE syringe filter to remove dust. Note that the filtration step is crucial as prolonged/repeated use of unfiltered stock solutions may lead to eventual clogging of the micromixer. As a secondary precaution to prevent clogging, we place 10 µm inline filters before the micromixer in our flow setup (see Supplementary Figure 2). The inline filters can be replaced with Luer lock syringe filters although this is not recommended when using high flow rates or when the flow system is operating under a large backpressure. The filtered PEO_{44}-*b*-$P4VP_{21}$-*b*-PS_{300} solution was then loaded into a rubber gasket-free syringe and degassed at least 3 times following the procedure in Supplementary Figure 3 before mounting the syringe onto a syringe pump. The water used was obtained directly from a Milli-Q dispenser and filtered through a 0.45 µm PES syringe filter prior to use. The required volume of water was loaded into a syringe and similarly degassed following the procedure in Supplementary Figure 3 for at least 5 times before mounting the syringe onto another syringe pump. Both syringes (one containing the organic phase and the other containing the aqueous phase) were then connected to the micromixer as shown in Supplementary Figure 2 to complete the continuous flow setup. Next, the syringe pumps were set to dispense at $Q_{organic}$ = 0.8 mL/min and Q_{water} = 0.2 mL/min (Q_{total} = 1 mL/min) to target the polymersome morphology. Once dispensing has begun, we typically allow the system to equilibrate for at least four times the volume of the equilibration loop (4 × 0.7 mL = 2.8 mL). After sufficient

equilibration, the resulting mixture was collected directly into a glass vial and gently swirled to ensure homogeneity. The sample was then allowed to age undisturbed or quenched by extensive dialysis against water in a 3.5 kDa MWCO dialysis tubing.

Continuous flow self-assembly procedure for PAA_{26}-*b*-PS_{81}

A 1 mg/mL stock solution of PAA_{26}-*b*-PS_{81} in THF was prepared beforehand and stirred in an airtight Schott bottle at 100 rpm until required. The same stock solution was used in all experiments to minimize any concentration errors. Whenever needed, the required volume of PAA_{26}-*b*-PS_{81} stock solution was removed with a rubber gasket-free syringe and filtered through a 0.45 µm PTFE syringe filter to remove dust. Note that the filtration step is crucial as prolonged/repeated use of unfiltered stock solutions may lead to eventual clogging of the micromixer. As a secondary precaution to prevent clogging, we place 10 µm inline filters before the micromixer in our flow setup (see Supplementary Figure 2). The inline filters can be replaced with Luer lock syringe filters although this is not recommended when using high flow rates or when the flow system is operating under a large backpressure. The filtered PAA_{26}-*b*-PS_{81} solution was then loaded into a rubber gasket-free syringe and degassed at least 3 times following the procedure in Supplementary Figure 3 before mounting the syringe onto a syringe pump. **Note:** the aqueous phase used in this experiment is 100 µM HCl solution instead of water as the PAA chains must be partially deprotonated in order to target the polymersome morphology. The 100 µM HCl solution used was directly filtered through a 0.45 µm PES syringe filter prior to use. The required volume of 100 µM HCl solution was loaded into a syringe and similarly degassed following the procedure in Supplementary Figure 3 for at least 5 times before mounting the syringe onto another syringe pump. Both syringes (one containing the organic phase and the other containing the aqueous phase) were then connected to the micromixer as shown in Supplementary Figure 2 to complete the continuous flow setup. Next, the syringe pumps were set to dispense at Q_{organic} = 4.4 mL/min and $Q_{100\ \mu\text{M HCl}}$ = 3.6 mL/min (Q_{total} = 8 mL/min) to target the polymersome morphology. Once dispensing has begun, we typically allow the system to equilibrate for at least four times the volume of the equilibration loop (4 × 0.71 mL = 2.84 mL). After sufficient equilibration, the resulting mixture was collected directly into a glass vial and gently swirled to ensure homogeneity. The sample was then allowed to age undisturbed or quenched by extensive dialysis against 100 µM HCl solution followed by water in a 3.5 kDa MWCO dialysis tubing. If necessary, prior to analysis by TEM and DLS, a drop of 0.1 M sodium hydroxide (NaOH) solution can be added to deprotonate the PAA chains on the polymersome surface, promoting chain repulsion and thus colloidal stability.

Continuous flow self-assembly and downstream annealing procedure for PEO_{44}-*b*-PS_{86}

A filtered PEO_{44}-*b*-PS_{86} solution was loaded into a 10 mL SGE gastight glass syringe and mounted onto a syringe pump. ***Note:*** the degassing step in Supplementary Figure 3 is not required in this setup because of the inclusion of a backpressure regulator (BPR) which prevents outgassing. Water was obtained directly from a Milli-Q dispenser and filtered through a 0.45 µm PES syringe filter prior to use. The required volume of water was loaded into another 10 mL SGE gastight glass syringe and mounted onto another syringe pump. Both syringes (one containing the organic phase and the other containing the aqueous phase) were then connected to the micromixer as shown in Supplementary Figure 8 to complete the continuous flow setup. We then submerge the 2 mL annealing loop (which gives a $t_{\text{residence,heating}}$ = 30 s at Q_{total} = 4 mL/min) into a water bath pre-heated to the desired temperature (20-70 °C). After 10 min of thermal equilibration, the syringe pumps were set to dispense at flow rates of Q_{organic} = 2.8 mL/min and Q_{water} = 1.2 mL/min (Q_{total} = 4 mL/min) to target the polymersome morphology. Once dispensing has begun, we typically allow the system to equilibrate for at least twice the volume of the sum of the equilibration loop and annealing loop (2 × (0.71 mL + 2 mL) = 5.42 mL). After sufficient equilibration, the resulting mixture was collected directly into a glass vial and then left to cool under ambient conditions (~10 min), before being quenched by extensive dialysis against water in a 3.5 kDa MWCO dialysis tubing.

Continuous flow self-assembly, downstream annealing, and downstream shape transformation procedure for PEO_{44}-*b*-PS_{86}

A filtered PEO_{44}-*b*-PS_{86} solution was loaded into a 10 mL SGE gastight glass syringe and mounted onto a syringe pump. ***Note:*** the degassing step in Supplementary Figure 3 is not required in this setup because of the inclusion of a backpressure regulator (BPR) which prevents outgassing. Water was obtained directly from a Milli-Q dispenser and filtered through a 0.45 µm PES syringe filter prior to use. The required volume of water was loaded into another 10 mL SGE gastight glass syringe and mounted onto another syringe pump. Both syringes (one containing the organic phase and the other containing the aqueous phase) were then connected to the micromixer as shown in Supplementary Figure 9 to complete the continuous flow setup. Note that a shut-off valve was placed between the syringe loaded with water and the micromixer to allow water to be replenished when necessary (n.b., the replenishment process can be performed as follows: (i) stop syringe pumps, (ii) set shut-off valve to "close" position, (iii) disconnect and replenish syringe with water, (iv) reconnect replenished syringe, (v) set shut-off valve to "open" position and (vi) start syringe pumps). The 2 mL annealing loop (which gives a $t_{\text{residence,heating}}$ = 30 s at Q_{total} = 4 mL/min) was submerged into a water bath pre-heated at 70 °C, while the 31.4 mL of cooling loop (which gives a $t_{\text{residence,cooling}}$ = 7 min 51 s at Q_{total} = 4 mL/min) was enclosed in an incubator pre-heated at 40 °C. Further down the line, a secondary micromixer was connected to the flow setup to introduce a stream of concentrated NaCl solution (c_{NaCl} = 5.05 M) via a syringe pump equipped with a plastic syringe (n.b., a gastight glass syringe is not required in this case because the system is no longer pressurized by the BPR at this point). After 10 min of thermal equilibration, the syringe

pumps were set to dispense at flow rates of $Q_{organic}$ = 2.8 mL/min and Q_{water} = 1.2 mL/min (Q_{total} = 4 mL/min) to target the polymersome morphology, and Q_{NaCl} = 0.04 mL/min to induce downstream shape transformation ($c_{NaCl,final}$ = 50 mM). We typically discard the first 8 mL of eluent to ensure the sample has been properly equilibrated prior to collection. In a typical experiment, we would collect and quench 5 mL of sample by extensive dialysis against water in a 3.5 kDa MWCO dialysis tubing.

Supplementary References

1. Wong, C. K., Mason, A. F., Stenzel, M. H. & Thordarson, P. Formation of non-spherical polymersomes driven by hydrophobic directional aromatic perylene interactions. *Nat. Commun.* **8**, 1240 (2017).
2. La, Y., An, T. H., Shin, T. J., Park, C. & Kim, K. T. A Morphological Transition of Inverse Mesophases of a Branched-Linear Block Copolymer Guided by Using Cosolvents. *Angew. Chem. Int. Ed.* **54**, 10483–10487 (2015).
3. Schneider, C. A., Rasband, W. S. & Eliceiri, K. W. NIH image to ImageJ: 25 years of image analysis. *Nat. Methods* **9**, 671–675 (2012).
